# Genome-wide *in silico* identification and characterization of the stress associated protein (SAP) gene family encoding A20/AN1 zinc-finger proteins in potato (*Solanum tuberosum L*.)

**DOI:** 10.1371/journal.pone.0273416

**Published:** 2022-08-23

**Authors:** Syyed Asim Billah, Nadir Zaman Khan, Waqar Ali, Muhammad Aasim, Muhammad Usman, Mohamed Amar Alezzawi, Habib Ullah

**Affiliations:** 1 Department of Biotechnology, University of Malakand, Chakdara, Khyber Pakhtunkhwa, Pakistan; 2 Department of Microbiology, University of Sebha, Sabha, Libya; University of Naples Federico II, ITALY

## Abstract

Stress associated proteins (SAPs) in plants have a key role in providing tolerance to multiple abiotic stresses. SAP gene family in *Solanum tuberosum* has not been fully studied before. This study identified 17 *StSAP* genes in *S*. *tuberosum* which code for A20/AN1 zinc-finger proteins. All the genes were distributed on ten different chromosomes and six segmental duplication events were identified. The SAPs in *S*. *tuberosum* and its orthologs in *Arabidopsis thaliana* were classified into six groups through the phylogenetic analysis. Introns across *StSAP* genes were identified in four genes. The promotor study of the *StSAP* genes showed different hormone and stress-related cis-elements that could potentially have a role in environmental stress response. The expression of *StSAP* genes in response to heat, mannitol, and salt were analyzed through *in silico* transcriptomic analysis. This study could potentially help in further understanding the functions of SAP genes in *S*. *tuberosum*.

## Introduction

Plants have developed different mechanisms of tolerating environmental stresses. Genetic regulation at the transcriptional level gives rise to morphological, biochemical and physiological changes in response to environmental stresses [1]. Stress-associated proteins (SAPs) gene family is identified that protects the plants against these environmental stresses. There are two zinc-finger domains present in SAP gene family, C-terminal A20 and N-terminal AN1 [2]. In a TNFα –inducible protein of human endothelial cells, the A20 zinc-finger domain was first identified. It regulates the immune response by inhibiting NFκB activity. There are multiple Cys_2_/ Cys_2_ finger motifs found in the A20 zinc-finger domain [3, 4]. In a ubiquitin-like fusion protein, the AN1 zinc-finger domain was first identified. *Xenopus laevis* animal hemisphere 1 (AN1) maternal RNA codes the ubiquitin-like fusion protein [5]. The yeast two-hybrid assay of *OsiSAP8* shows that AN1 and A20 domains interact with each other [6] and hence, they are generally associated with each other [7].

In plants and animals, proteins having A20-AN1 zinc-finger domains provide tolerance to environmental stresses. In animals, a zinc-finger protein ZNF216 regulates immune response while the same function is performed by AWP1 in humans [8]. Zinc-finger proteins in plants help them tolerate different abiotic stresses. OsiSAP1, a protein having A20-AN1 domains was identified in Indica rice. In rice, *OsiSAP1* provides tolerance to cold stress, dehydration, salt, waterlogging, heavy metals, injury, and abscisic acid (ABA). The overexpression of *OsiSAP1* in tobacco and rice could potentially provide tolerance to water-deficit stress [9, 10]. Other *OsSAP* genes are also involved in abiotic environmental stress responses as they are triggered by one or more stresses [11]. It is demonstrated experimentally that the over-expression of *OsiSAP8* provides tolerance to low temperature, water deficit, and salinity stresses [6]. Stress associated proteins in *A*. *thaliana* significantly help in providing tolerance to different environmental stresses as well. e.g. *AtSAP5* is expressed in response to cold and plant growth hormones [12] and regulates the expression of heat-responsive genes [13]. In *A*. *thaliana*, overexpressing *AtSAP10* provides tolerance to heat and heavy metal stresses [14]. Besides Arabidopsis and rice, SAPs are also found in other plants and have an integral role in different stress responses [14]. In *Sorghum bicolor*, *SbSAP14* is induced by drought, salinity, and oxidative stress [15]. *MusaSAP1* in bananas helps in enduring various stresses [16].

*S*. *tuberosum* (Potato) is a member of the Solanaceae family and Solanum genus. Potato is a root vegetable and a useful source of energy and makes up a large portion of the world’s food stock after maize, wheat, and rice. Potato plants are perennials and they have about 5,000 varieties around the world, and it is a tetraploid with 48 chromosomes. As potatoes encounter numerous abiotic stresses, it is important to develop certain potato cultivars that are resistant to various stresses. As SAPs play a significant role in environmental abiotic stress response and for better understanding the molecular mechanism of the stress response, the identification and distribution of SAPs in potatoes is important.

## Methods

### Identification, characteristics and sequences of SAPs in *S*. *tuberosum*

Stress-associated proteins are characterized by having two main domains e.g. A20 (Prosite: PS51036) and AN1 (Prosite: PS51039). Using these IDs as keywords, SAPs were identified in *S*. *tuberosum* using the UniProt database [17] (https://www.uniprot.org/).

Ensembl Plant database (https://plants.ensembl.org/index.html) was used to download the genomic, CDS, Protein sequences and GFF files of the stress associated protein gene family. Other important information like transcript IDs, nucleotide length, chromosome number, and genomic coordinates were also downloaded from this database.

Using ProtParam tool (https://web.expasy.org/protparam/), different chemical and physical properties of StSAPs amino acids sequences were predicted such as; protein length, molecular weight, theoretical pI, and grand average of hydropathicity (GRAVY) [18].

### Phylogenetic analysis, sequence alignment and conserved motif identification

MEGA X software was used to undertake phylogenetic analysis [19]. Multiple sequence alignments were created of the Potato and Arabidopsis SAP protein sequences using Clustal W in MEGA X. The Neighbor-Joining approach was used to generate the tree with 500 bootstrap replications. For the alignment of protein sequences Clustal Omega were used (https://www.ebi.ac.uk/Tools/msa/clustalo/). The data generated by Clustal Omega was used in the box shade for conserved regions identification (https://embnet.vital-it.ch/software/BOX_form.html). Prosite was used to generate the predicted domain’s location (https://prosite.expasy.org/mydomains/).

### Gene location, duplication, and structure analysis

The *StSAP* gene location on chromosomes [20] was demonstrated by using a web-based Phenogram (http://visualization.ritchielab.org/phenograms/plot). Using Gene Structure Display Server (GSDS) (http://gsds.gao-lab.org/), the exon-intron structure was analyzed. Circos map was constructed using TBtools. The required input files for making an advanced Circos map were created from the saved data. Consequently, the members of the StSAP family were placed in different groups and a specific color was given to each group.

Using TBtools [21], the Non-synonymous and synonymous mutation values were calculated. The calculation for both the values required a gene pair, CDS, and protein FASTA sequences which were used as queries in the simple Ka/Ks calculator in the Tbtools. Furthermore, the calculation for duplication time (*T*) was performed using the formula T = Ks/2λ × 10^−6^ Mya (λ = 2.6 × 10^−9^) [22].

### Collinearity analysis

TBtools was used for the collinearity analysis between the SAPs of *A*. *thalian* and *S*. *tuberosum*. For the collinearity analysis, the genomic files and GFF3 files of *S*. *tuberosum* and *A*. *thaliana* were used to obtain the data that was later used to show the relationship between the stress associated proteins of both the species.

### Protein-protein interaction analysis

STRING (Search Tool for the Retrieval of Interacting Genes/Proteins) was searched by single protein name of all the SAP proteins [23]. The transcript ids were used as a query and selecting *Solanum tuberosum* in the organism field. By narrowing down the predicted functional partners, highest combine confidence score (≥ 0.8) is selected. The protein possible name and GO (gene ontology) annotations were extracted from Uniport database.

### *In silico* transcriptome analysis

Potato eFP browser (https://bar.utoronto.ca/efp_potato/cgi-bin/efpWeb.cgi) was used for the *in silico* transcriptome analysis of *StSAP* genes. The expression data of each *SAP* gene was obtained from the eFP browser database [24]. A heatmap was constructed in the TBtools that showed the expression of *SAP* genes in response to certain stresses.

### *Cis*-elements analysis of StSAP genes

Using PlantCARE database (http://bioinformatics.psb.ugent.be/webtools/plantcare/html/), the promoter regions of the StSAP genes [25] revealed certain cis-acting regulatory elements. Using the Ensembl Plant database, upstream of the transcriptional start site, 1500 bp of genomic DNA sequence was retrieved and analyzed by PlantCARE.

## Results

### Identification of stress-associated proteins in the *S*. *tuberosum* genome

Stress-associated proteins were identified in *S*. *tuberosum* genome using the Uniprot database. Prosite identifiers of A20 (PS51036) and AN1 (PS51039) domains were used as keywords. A total of 21 proteins were recognized that had A20-AN1 domains. The redundant proteins were removed and the overall number was reduced to 17. Using the Prosite database, the presence of A20-AN1 domains was confirmed in each protein using the amino acid sequence. SAP genes in *S*. *tuberosum* were distributed on ten different chromosomes (Table 1).

**Table 1:** Genes coding stress-associated proteins in *S*. *tuberosum*.

Gene ID	Transcript ID	Name	Chromosome	CDS (bp)	Location	Stand
PGSC0003DMG400019467	PGSC0003DMT400050131	*StSAP6*	11	979	5,167,017–5,167,995	Reverse
PGSC0003DMG400003948	PGSC0003DMT400010084	*StSAP1*	8	1202	53,398,986–53,400,187	Forward
PGSC0003DMG400028167	PGSC0003DMT400072393	*StSAP3*	10	948	55,736,220–55,737,167	Reverse
PGSC0003DMG400002697	PGSC0003DMT400006957	*StSAP4*	9	1020	3,090,742–3,091,761	Reverse
PGSC0003DMG400006814	PGSC0003DMT400017570	*StSAP5A*	1	1505	66,086,408–66,087,912	Forward
PGSC0003DMG400031871	PGSC0003DMT400081478	*StSAP2A*	1	607	49,228,441–49,229,047	Reverse
PGSC0003DMG400008152	PGSC0003DMT400021060	*StSAP2C*	10	1069	58,643,228–58,645,856	Forward
PGSC0003DMG400006813	PGSC0003DMT400017568	*StSAP2D*	1	1080	66,071,086–66,075,234	Forward
PGSC0003DMG400013600	PGSC0003DMT400035389	*StSAP2B*	11	1342	34,058,526–34,061,978	Reverse
PGSC0003DMG400047330	PGSC0003DMT400097759	*StSAP10A*	7	408	50,231,496–50,231,903	Reverse
PGSC0003DMG400036158	PGSC0003DMT400086587	*StSAP10B*	7	375	50,211,120–50,211,494	Reverse
PGSC0003DMG400037614	PGSC0003DMT400088043	*StSAP10C*	7	408	50,227,416–50,227,823	Reverse
PGSC0003DMG400031013	PGSC0003DMT400079636	*StSAP7*	3	1347	41,010,353–41,018,084	Forward
PGSC0003DMG400023837	PGSC0003DMT400061226	*StSAP5B*	12	770	60,714,780–60,715,549	Reverse
PGSC0003DMG400023684	PGSC0003DMT400060887	*StSAP11*	10	1224	57,695,732–57,699,608	Forward
PGSC0003DMG400027241	PGSC0003DMT400070044	*StSAP13*	4	1336	9,908,680–9,912,818	Forward
PGSC0003DMG400000512	PGSC0003DMT400001376	*StSAP12*	2	944	46,764,282–46,765,379	Reverse

Chromosomes 7 and 10 had 3 genes each, while chromosomes 1 and 11 had 2 genes each. Each of the remaining chromosomes had only 1 gene. For each StSAP gene, the transcript length was different and found to be between 375 and 1505 bp. Physiochemical analysis of StSAPs revealed that the amino acid length of each protein ranged from 124 to 448 amino acids (aa), the molecular weight of each protein was found to be different and ranged from 14.25 to 50.05 kDa, PI ranged from 6.79 to 9.26. Likewise, the GRAVY values were negative for each protein which illustrated the hydrophilic nature of StSAPs. The analysis of the subcellular location revealed that most of the StSAPs were present in the nucleus (Table 2).

**Table 2:** Distribution of StSAP protein family containing A20/AN1 domains.

Protein ID	Protein Name	Zinc-finger	Protein Length	Mol wt (kDa)	pI	GRAVY	Subcellular Localization
PGSC0003DMG400019467	StSAP6	A20-AN1	183	20.34221	8.85	-0.628	Nucleus
PGSC0003DMG400003948	StSAP1	A20-AN1	166	17.82851	8.83	-0.25	Cytosol
PGSC0003DMG400028167	StSAP3	A20-AN1	159	17.11878	8.33	-0.157	Cytosol
PGSC0003DMG400002697	StSAP4	A20-AN1	150	16.86476	8.87	-0.335	Cytosol
PGSC0003DMG400006814	StSAP5A	A20-AN1	187	20.8748	9.12	-0.688	Nucleus
PGSC0003DMG400031871	StSAP2A	A20-AN1	171	18.2959	6.79	-0.357	Nucleus
PGSC0003DMG400008152	StSAP2C	A20-AN1	172	18.22471	8.47	-0.397	Nucleus
PGSC0003DMG400006813	StSAP2D	A20-AN1	167	17.96438	7.47	-0.283	Nucleus
PGSC0003DMG400013600	StSAP2B	A20-AN1	170	18.2628	8.46	-0.372	Nucleus
PGSC0003DMG400047330	StSAP10A	A20-AN1	135	15.11645	9.01	-0.525	Nucleus
PGSC0003DMG400036158	StSAP10B	A20-AN1	124	14.25755	8.93	-0.479	Nucleus
PGSC0003DMG400037614	StSAP10C	A20-AN1	135	15.15247	8.59	-0.423	Nucleus
PGSC0003DMG400031013	StSAP7	A20-AN1	448	50.05012	9.26	-0.75	Nucleus
PGSC0003DMG400023837	StSAP5B	AN1	159	17.54701	9.26	-0.689	Nucleus
PGSC0003DMG400023684	StSAP11	AN1-AN1-C2H2_2	272	30.28527	8.63	-0.642	Nucleus
PGSC0003DMG400027241	StSAP13	AN1-AN1-C2H2_2	277	31.40977	8.7	-0.684	Nucleus
PGSC0003DMG400000512	StSAP12	AN1-AN1	187	20.77983	8.85	-0.607	Nucleus

### Phylogenetic analysis of StSAPs

**Fig 1:**
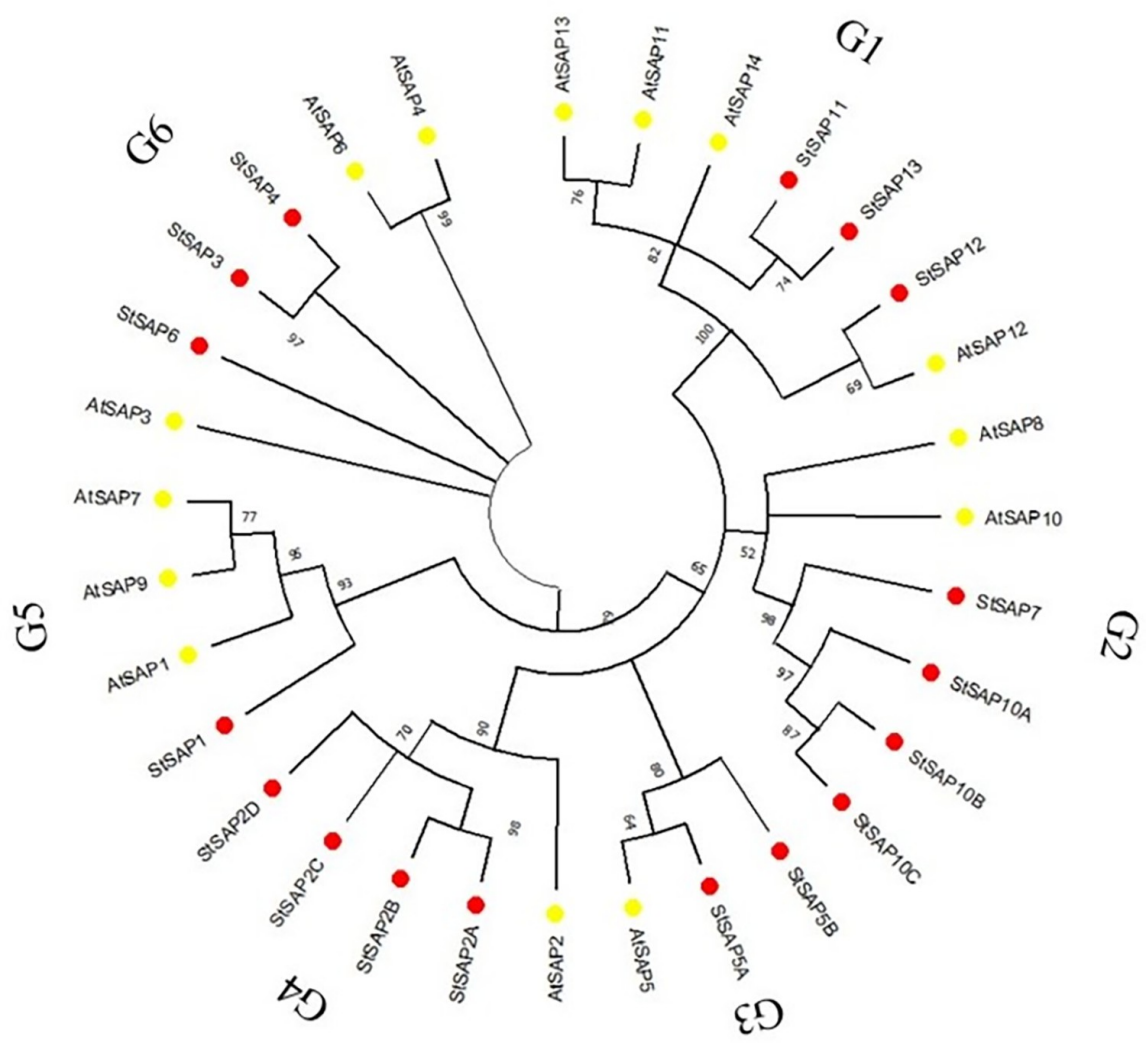
Phylogenetic tree showing SAPs in *S*. *tuberosum* (Red) and *A*. *thaliana* (Yellow).

The amino acid sequences of stress-associated proteins from *A*. *thaliana* and *S*. *tuberosum* were used to create the phylogenetic tree. The phylogenetic tree showed that SAPs from both the *S*. *tuberosum* and *A*. *thaliana* were grouped into six groups (Fig 1). Group 1 contained AtSAP11, AtSAP12, AtSAP13, AtSAP14, StSAP11, StSAP12, and StSAP13. Group 2 had AtSAP8, AtSAP10, StSAP7, StSAP10A, StSAP10B, StSAP10C. AtSAP5, StSAP5A, and StSAP5B constituted group 3. Group 4 contained AtSAP2, StSAP2A, StSAP2B, StSAP2C, and StSAP2D. Group 5 had AtSAP1, StSAP1, AtSAP7, and AtSAP9. The last group consisted of AtSAP3, StSAP3, AtSAP4, StSAP4, AtSAP6, and StSAP6.

### Identification of conserved motifs in the SAP family

**Fig 2:**
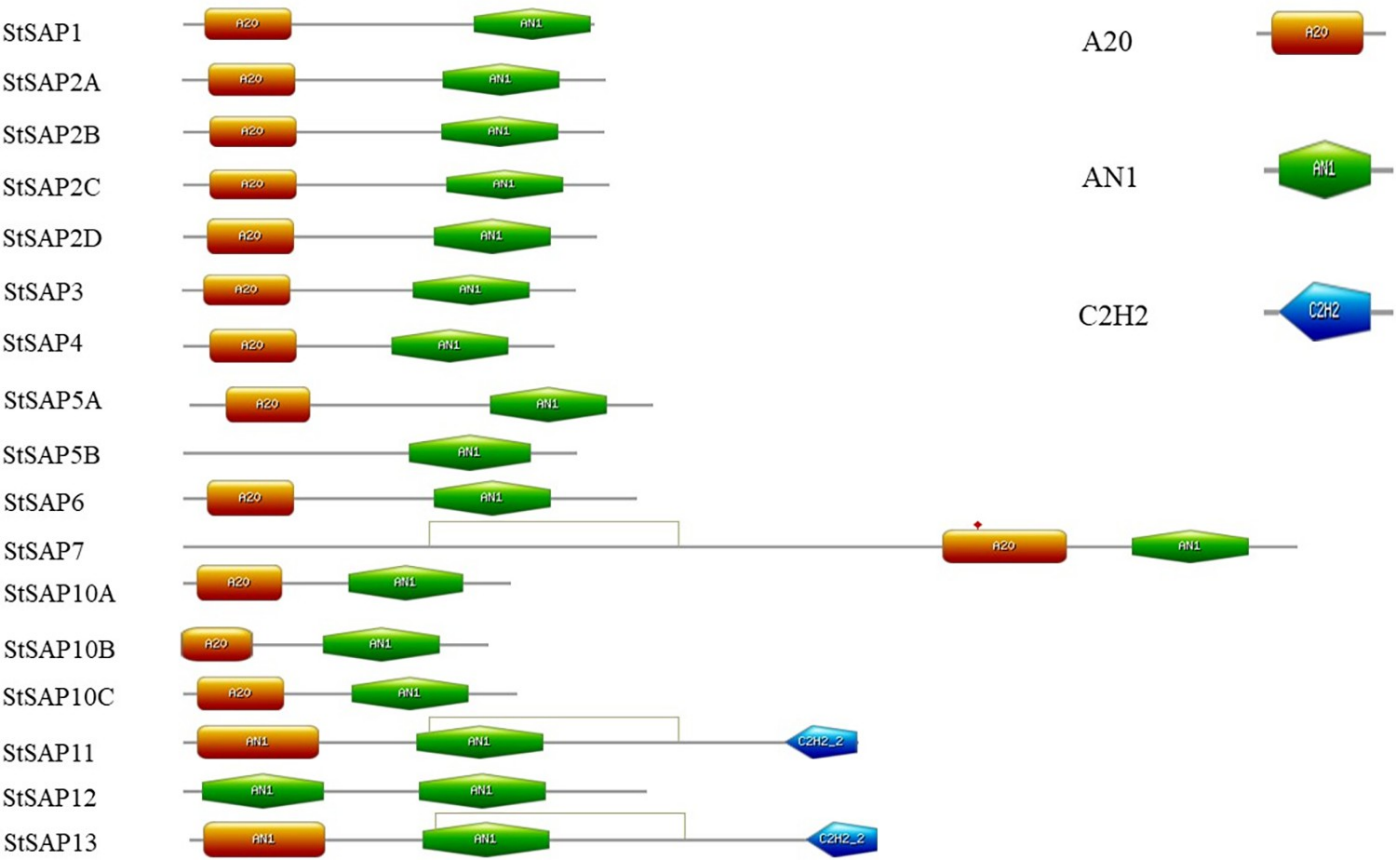
Distribution of conserved motifs in *S*. *tuberosum* SAP family.

The conserved domains of the SAP family in *S*. *tuberosum* were identified in Prosite database, which is also supported by multiple sequence alignment and almost all the proteins had both A20-AN1 domains (Fig 2 and S1 Fig). StSAP5B had only one AN1 domain, StSAP12 had two AN1 domains, and StSAP11 and StSAP13 had the same arrangement as both contained two AN1 and one C2H2 domain. The length and location of the domains were determined.

### Selective pressure analysis of SAP genes

The calculation of the Ka/Ks value for each pair of the duplicated genes estimated the rate of evolution. For all the duplicated *StSAP* genes during the duplication events, purifying selection pressure was determined by Ka/Ks values less than 1 (Table 3). The purifying selection pressure illustrated that in the subsequent evolutionary process, the function of the *StSAP* gene might have remained the same.

**Table 3:** Estimated time for segmental duplication and Ka/Ks values for *StSAP* genes.

Seq_1	Seq_2	Ka	Ks	Ka/Ks	T(MYA)
StSAP2A	StSAP2B	0.131859	0.702207	0.187778	135.0397296
StSAP2C	StSAP2D	0.223636	1.86313	0.120032	358.2942178
StSAP3	StSAP4	0.281861	1.96248	0.143625	377.400065
StSAP11	StSAP13	0.132	0.699649	0.188666	134.5479687
StSAP10B	StSAP10C	0.051515	0.088639	0.581176	17.04592005

### Structural divergence of *StSAP* genes

**Fig 3:**
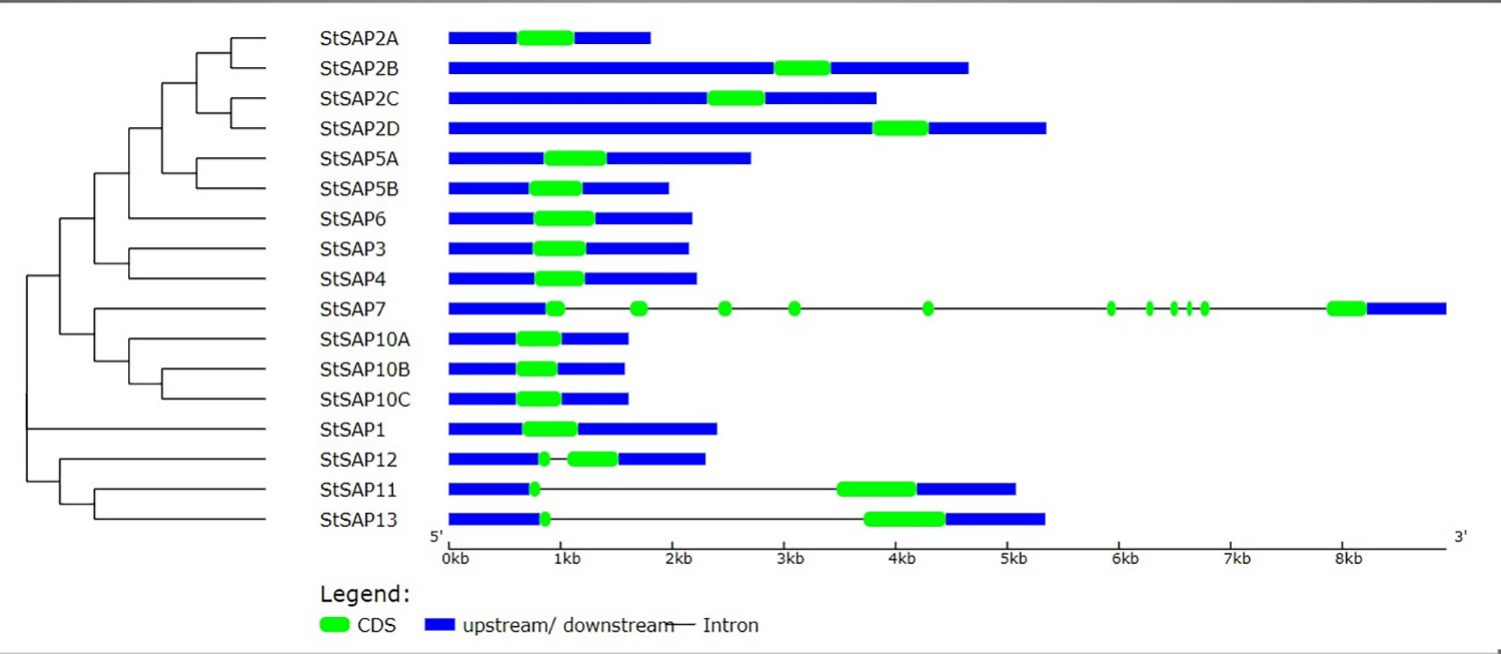
The distribution of exon-intron in the structure of *StSAP* genes according to the phylogenetic relationship.

A comparison between CDS and genomic DNA sequences was performed for each *StSAP* gene to understand the structural divergence among the *StSAPs* genes. Many genes contained only one exon (Fig 3), while *StSAP11*, *StSAP12*, and *StSAP13*, each contained two exons and one intron and were included in group 5. *StSAP7* had 11 exons and one intron and was found in group 4.

### Chromosomal distribution and duplication of *StSAP* genes

**Fig 4:**
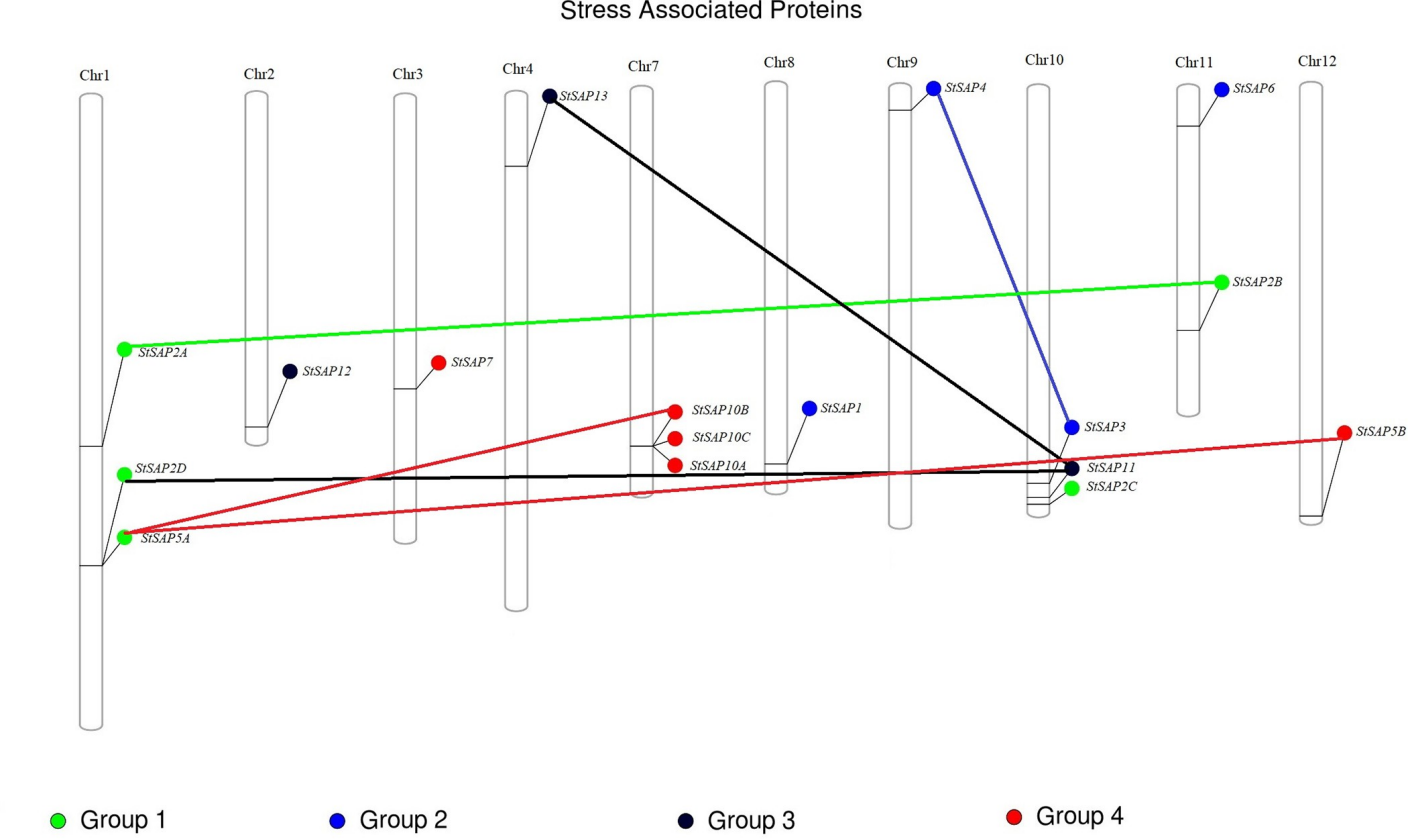
The presence of *StSAP* genes on *S*. *tuberosum* chromosomes. The lines connecting the genes show segmental duplication.

The location of the *StSAP* genes on different chromosomes was studied using a web-based phenogram. It was found that the *StSAP* genes were distributed on 10 separate chromosomes (Fig 4). *StSAP2A*, *StSAP2D*, and *StSAP5A* were found on chromosome 1. *StSAP12* was present on chromosome 2 while *StSAP7* was found on chromosome 3. Chromosome 4 had *StSAP13*. *StSAP10A*, *StSAP10B*, and *StSAP10C* were present on chromosome 7. *StSAP1* was found on chromosome 8, and *StSAP4* was present on chromosome 9. *StSAP2C*, *StSAP3*, and *StSAP11* were located on chromosome 10. *StSAP2B* and *StSAP6* were located on chromosome 11 and *StSAP5B* was located on chromosome 12. The duplicated genes suggest that these genes evolved from the same parent gene. The Circos map drawn by the TBtools also confirmed 5 pairs of segmental duplication gene pairs (S2 Fig). *StSAP2A* was linked with *StSAP2B*, *StSAP5A* had linkage with *StSAP10B* and *StSAP5B*. *StSAP11* was linked with *StSAP2D* and *StSAP13*. Finally, *StSAP3* showed linkage with *StSAP4*.

### Collinearity analysis of *StSAPs* and *AtSAPs*

**Fig 5:**
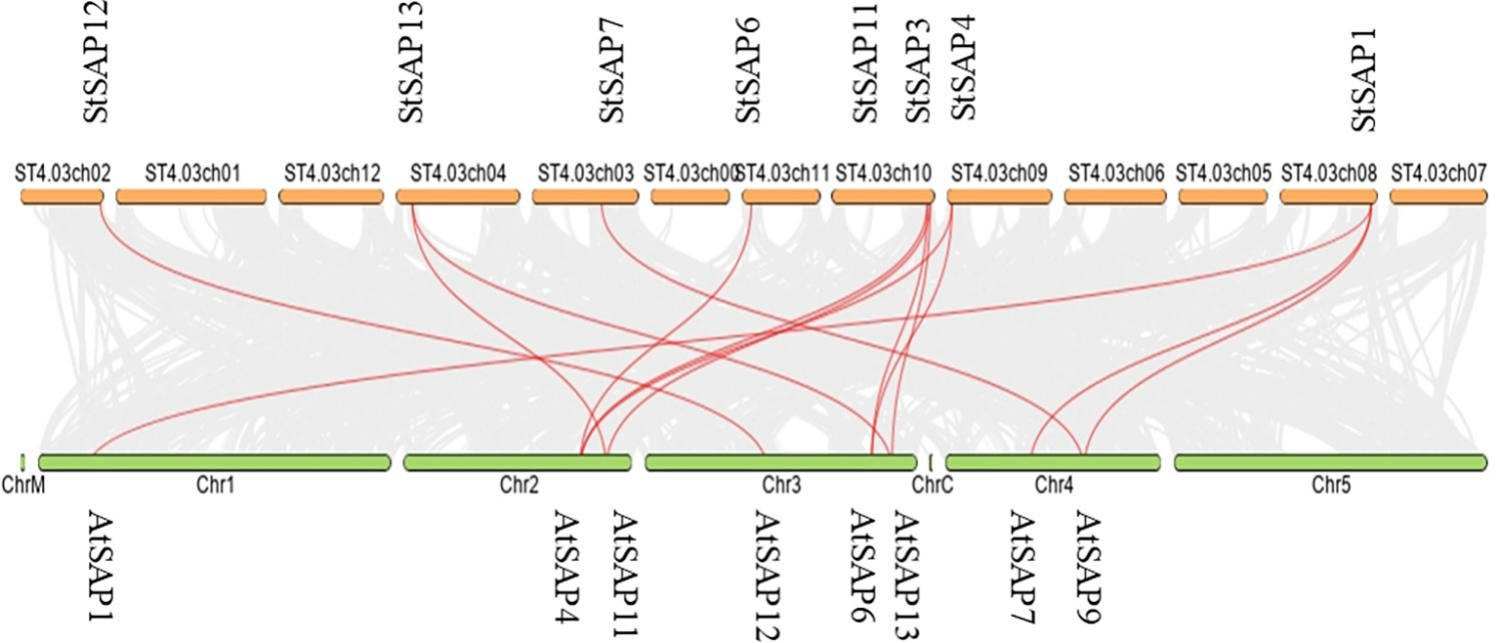
Synteny analysis between *S*. *tuberosum* and *A*. *thaliana SAP* genes. The chromosomes are represented by the horizontal bars and the curves show the linkage among the genes.

The relation between the *S*. *tuberosum* and *A*. *thaliana* SAP genes was studied by the synteny analysis between their genomes (Fig 5). *StSAP1* gene located on chromosome 8 was associated with three genes from Arabidopsis, namely, *AtSAP1*, *AtSAP9*, and *AtSAP7*. Similarly, *StSAP4* gene found on chromosome nine was related to two genes from Arabidopsis, viz. *AtSAP4* and *AtSAP6*. *StSAP3* and *AtSAP6* were found to be related to each other. Likewise, *StSAP11* was associated with *AtSAP11* and *AtSAP13*. *StSAP6* was linked with *AtSAP4*, *StSAP7* with *AtSAP9*, and *StSAP13* was linked with *AtSAP13* and *AtSAP11*. *StSAP12* and *AtSAP12* were observed to be linked with each other.

### Protein-protein interaction analysis

STRING database is used to search the possible protein interactors of the SAP proteins in potato (Table 4). The interactome analysis revealed that most of the SAP proteins except SAP11, SAP12 and SAP13 showed a strong interaction with three proteins (PGSC0003DMT400019238, PGSC0003DMT400050774, PGSC0003DMT400055259). All the three interactors contain a potential RAB GTPase domain, which indicates that these are RAB GTPase proteins. The remaining SAP Proteins (SAP11,12,13) showed interaction with NPL4 (nuclear protein localization homolog 4) family protein (PGSC0003DMT400061780).

**Table 4:** Potential protein interactor partners of the stress associated proteins (SAP) using STRING database.

SAP	Interactor ID	Name (Uniprot)	GO (Gene Ontology) Annotations
StSAP1 StSAP2A StSAP2B StSAP2C StSAP2D StSAP3 StSAP4 StSAP5A StSAP5B StSAP6 StSAP7 StSAP10A StSAP10B StSAP10C	PGSC0003DMT400019238 PGSC0003DMT400050774 PGSC0003DMT400055259	Rab related proteins	Cellular Component: endosome membrane, plasma membrane Molecular function: GTP binding, GTPase activity. Biological process: endocytic recycling, Intracellular protein transport.
StSAP11 StSAP13 StSAP12	PGSC0003DMT400061780	NPL4 family protein	Cellular Component: nucleus, Molecular function: ubiquitin binding Biological process: ubiquitin-dependent protein catabolic process

### Analysis of regulatory elements in promoters of *StSAP* genes

Regulatory elements in *StSAP* promoter sequences were analyzed to understand the regulation patterns and gene function. Consequently, 14 different elements were identified in the *StSAP* promoters that were stress and hormone-responsive (Table 5). Among the 14 elements, the stress-responsive regulatory elements identified were LTR elements, the MYB transcription factor, ARE and Box-W1. The identified hormone-related cis-elements were the TGA element, AuxRR core, ABRE, P-box, GARE motif, TCA element, CGTCA motif and TGACG motif. TCA-rich repeats and MYB transcription factor was found in most of the *StSAP* promoters. The GARE motif was only found in the *StSAP2A* promoter while the AuxRR core was present in *StSAP4*, *StSAP6*, and *StSAP10B*.

**Table 5:** Regulatory elements in the promoter regions of StSAP genes.

Genes	TC rich repeats	LTR	MBS	ARE	Box W1	TGA element	AuxRR core	ABRE	P Box	GARE motif	TCA element	CGTCA motif	TGACG motif	MYB
*StSAP1*	1							6	3			2		
*StSAP2A*	1	2		2	1	1		3	1	1	2			5
*StSAP2B*	1		1	3	1				1			4		5
*StSAP2D*	1				3	1			2		2		3	7
*StSAP3*			1	2				3			1	2		2
*StSAP4*							1	1	2					7
*StSAP5B*						2		3			1			
*StSAP6*						1	1	1						4
*StSAP7*	1	1	1	2								1	1	1
*StSAP10A*			1					1	1					
*StSAP10B*		2					1	2	1				3	2
*StSAP10C*	1	2		2				3	1			3	3	2
*StSAP11*	1					1					1	1		2
*StSAP12*	1		1					2	1		1	3	3	5
*StSAP13*	2		2						1					4

### Transcriptional responses of *StSAP* genes to abiotic stresses

**Fig 6:**
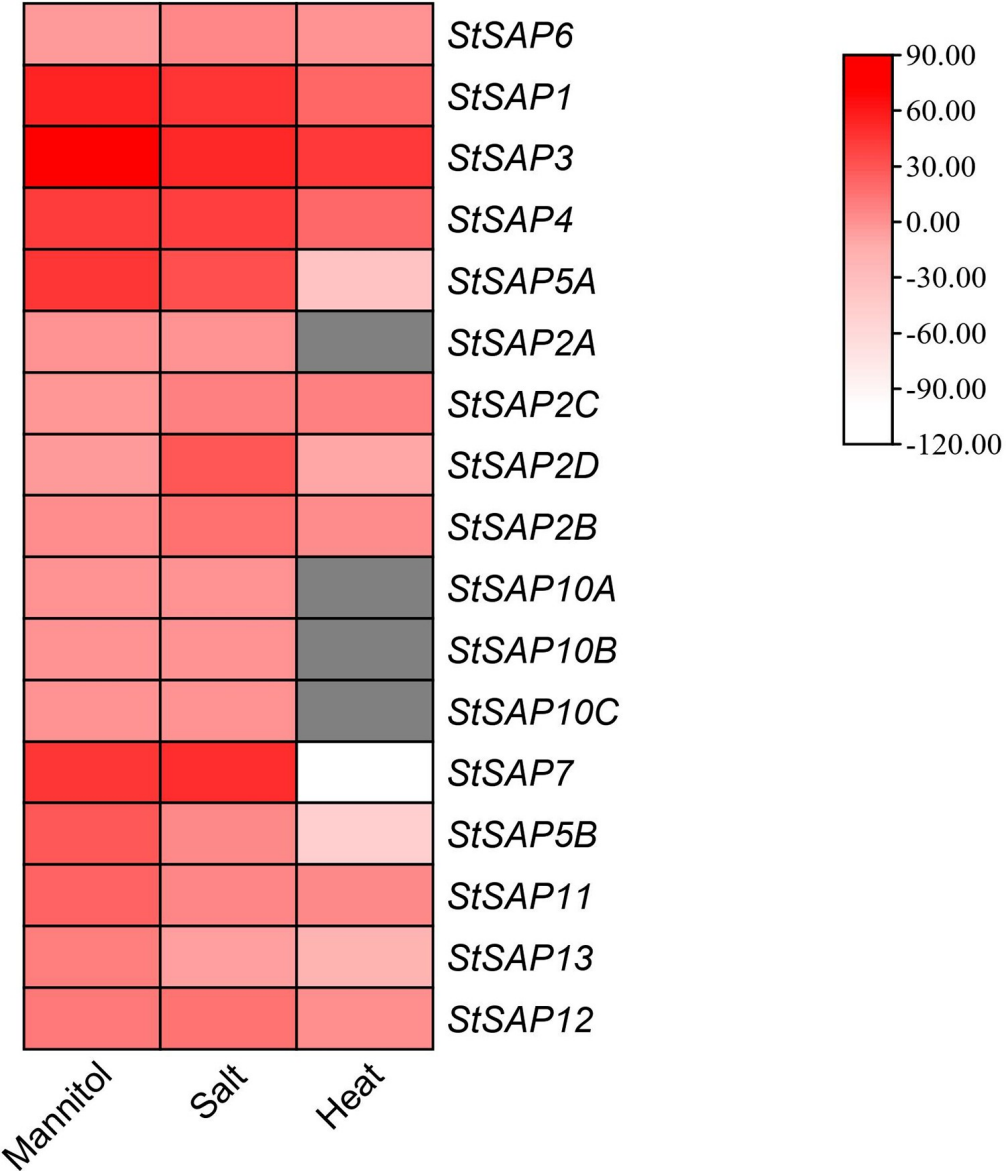
Expression analysis of *StSAP* genes under mannitol, salt, and heat.

*In silico* transcriptome analysis was performed using the Potato eFP browser. The analysis showed the upregulation or downregulation of the *StSAP* genes in the presence of certain abiotic stresses. Consequently, upregulation was observed in the expression of almost all the *StSAP* genes in the presence of salt, heat, and mannitol stresses (Fig 6). The expression of *StSAP2D*, *StSAP5A*, *StSAP5B*, *StSAP7*, and *StSAP13* had a lower expression in the presence of heat stress. *StSAP3* was upregulated in the presence of heat, stress, and mannitol stresses. Salt stress downregulated the expression of *StSAP13*. *StSAP6*, *StSAP2C*, and *StSAP2D* were downregulated in the presence of mannitol stress. The expression of *StSAP2A*, *StSAP10A*, *StSAP10B*, *and StSAP10C* remained the same in the presence of all three stresses.

## Discussion

Stress-associated proteins (SAPs) in plants play a pivotal part in generating tolerance to various abiotic stresses. This is an extensive study on the SAP gene family in different plants. Here, we recognized 17 SAP proteins and their respective genes in *S*. *tuberosum* using UniProt and Ensembl Plant databases. SAP genes play an important role in stress responses, for instance, In *India* rice, the first gene *OsiSAP1* was characterized for its function [10]. The number of SAP genes is diverse among various plants. For example, SAP genes identified in Arabidopsis are 14 [26], rice has 18 [26], the tomato has 13 [27], maize has 11 [28], *Populus euphratica* has 18 [29], *P*. *trichocarpa* contains 19 [28], 19 in *Salix purpurea* [29], and *S*. *suchowensis* has 15 [29], and cotton contains 37 [30]. The 17 genes identified in *S*. *tuberosum* code for proteins that contain A20 and AN1 domains. Almost all of the StSAPs have both domains i.e. A20 and AN1. which are generally related to each other. In *S*. *tuberosum*, StSAP5B had only one AN1 domain while StSAP12 had two AN1 domains. There are two AN1 and one C2H2 domain present in StSAP11 and StSAP13. Interestingly, in rice, there are two AN1 and two C2H2 domains present in OsSAP16 while *OsSAP18* has only an A20 domain. OsSAP13, OsSAP14, and OsSAP15, each has one AN1 domain. In Arabidopsis, *AtSAP14* has only AN1 domain while *AtSAP12* has two AN1 domains. *AtSAP11* contains two AN1 and two C2H2 domains and *AtSAP13* has two AN1 and one C2H2 domain [26]. The different physicochemical properties of SAP genes in *S*. *tuberosum* were studied and it was found that the SAP in Arabidopsis had the similar range of properties, such as amino acid and nucleotide length.

The phylogenetic relationship between *S*. *tuberosum* SAPs and *A*. *thaliana* SAPs was analyzed. The SAPs proteins from both species were placed into 6 groups. There were highly conserved domains present in proteins that were in the same group, suggesting they might be similar in functions.

The alterations in exon-intron structure have a pivotal role in the gene families’ evolution. Here, most *StSAP* genes had similar structures which suggested that these genes were highly structurally conserved. Like most plants, the SAP gene family in *S*. *tuberosum* revealed that most of them had no introns. Only 4 genes had introns while the rest were intron free. In *M*. *truncatula* most of the SAP genes have intron which is rarely observed [2]. The structure of SAP genes in rice shows that 11 genes have zero introns and 6 genes have one intron. However, there are two introns present in *OsSAP8*. In Arabidopsis, no introns have been found in 9 *AtSAPs* and 4 genes have only 1 intron while three introns are found in *AtSAP14* [26]. Under abiotic stresses, SAP genes that do not have introns are rapidly transcribed and translated which reduces posttranscriptional processing [31].

Segmental and tandem duplication plays a pertinent role in generating gene families in the process of evolution [32]. The strength and mode of natural selection act on protein-coding genes are determined by the Ka/Ks values. A positive selection effect occurs when the Ka/Ks values are higher than 1. Purifying selection is observed when the values are lower than 1 and values equal to 1 determine neutral selection [33].

Genes duplicate when living organisms evolve with time. In this way, they give rise to genes with different structures and functions. While studying SAPs in *S*. *tuberosum*, 10 out of 17 SAPs were likely to be duplicated genes. These genes had similar gene structures and coded for the same zinc-finger domains. For instance, StSAP13 and StSAP11 had only one intron and coded for three types of zinc-finger domains (A20-AN1-C2H2). The collinearity analysis between the *A*. *thaliana* and *S*. *tuberosum* showed SAP genes that were linked and they are also similar to each other structurally and functionally. For example, AtSAP11 and AtSAP13 both code for the same domains as their orthologs in *S*. *tuberosum*. This collinearity and structure similarity between both species suggest that orthologs in potato might be involved in different environmental stresses.

Evidence shows that in the presence of various abiotic stresses, the expression of SAP genes increases in different plants. For instance, *AtSAP5* [12], *AtSAP10* in *A*. *thaliana* [14], *SbSAP14* in *Sorghum bicolar* [15], *MusaSAP1* in banana [16], *OsiSAP1* and [9] *OsiSAP8* in rice [6] plays a key role in environmental stress response. In this study, In-silico transcriptome analysis revealed almost all the *StSAP* genes upregulated or downregulated in the presence of salt, mannitol, and heat stresses. While studying the promoters of *StSAP* genes, certain cis-elements were identified: TC-rich repeat works as a stress-responsive and defense element, LTR elements respond during lower temperature, MYB works during drought response (MBS), Box-W1 and ARE in the presence of heat stress. AuxRR core and TGA element are involved in auxin response, ABA and ABRE are abscisic acid-responsive elements, P Box and GARE motif are involved in gibberellin response and in salicylic acid (SA) response, TCA elements are involved. CGTCA and TGACG are functional in the presence of methyl jasmonate (MeJA). Like other species SAP family, changes in expression level under different stresses and the presence of various stress regulated cis elements confirmed StSAP role in abiotic stresses. Moreover Rab GTPases, the interacting partners with SAP proteins have one of the main roles in vesicle trafficking. The available literature suggests that Rab GTPases have diverse functional role in various cellular processes including plant growth and development, autophagy, plant microbe interactions, biotic and abiotic stresses [34]. NLP4 family proteins, other protein interactor with SAP11, 12 and 13, is integral component of highly conserved chaperone complex (Ufd1- Npl4-p97) and plays an important role in Endoplasmic reticulum associated degradation (ERAD). During ERAD the abnormal or misfolded proteins accumulated during extreme environmental conditions are recognized by Ufd1- Npl4-p97 chaperone complex and delivered to proteosome complex for processing [35].

In conclusion, a total of 17 genes were identified which were distributed on 10 different chromosomes. Based on the *In-silico* promoter, transcriptome and protein-protein interaction analysis, it is concluded that the SAP gene family could be involved in response to different abiotic stresses. This study will help in enhancing our understanding about the functions of SAP gene family in *S*. *tuberosum*. For further characterization of the *StSAPs*, experimental evidence is required to further understand their biological functions.

## Supporting information

S1 Fig Alignment of the stress-associated proteins in *S*. *tuberosum* performed using Clustal Omega. A20 zinc-finger domains are characterized by conserved cysteine residues and labelled Red, whereas the AN1 zinc-finger domain has conserved histidine and cysteine residues and colored Green. Proteins that had C2H2 zinc-finger domains were colored Pink.
(TIF)

S2 Fig The Circos map created via the TBtools shows the segmental duplication of the *StSAP* gene family in *S*. *tuberosum*. (TIF)
