# Supplementary figures and images for: Genome-wide in silico identification and characterization of the stress associated protein (SAP) gene family encoding A20/AN1 zinc-finger proteins in potato (Solanum tuberosum L.)
Source: PLoS One. 2022 Aug 23;17(8):e0273416. doi: 10.1371/journal.pone.0273416 (PMC9398024; doi:10.1371/journal.pone.0273416)

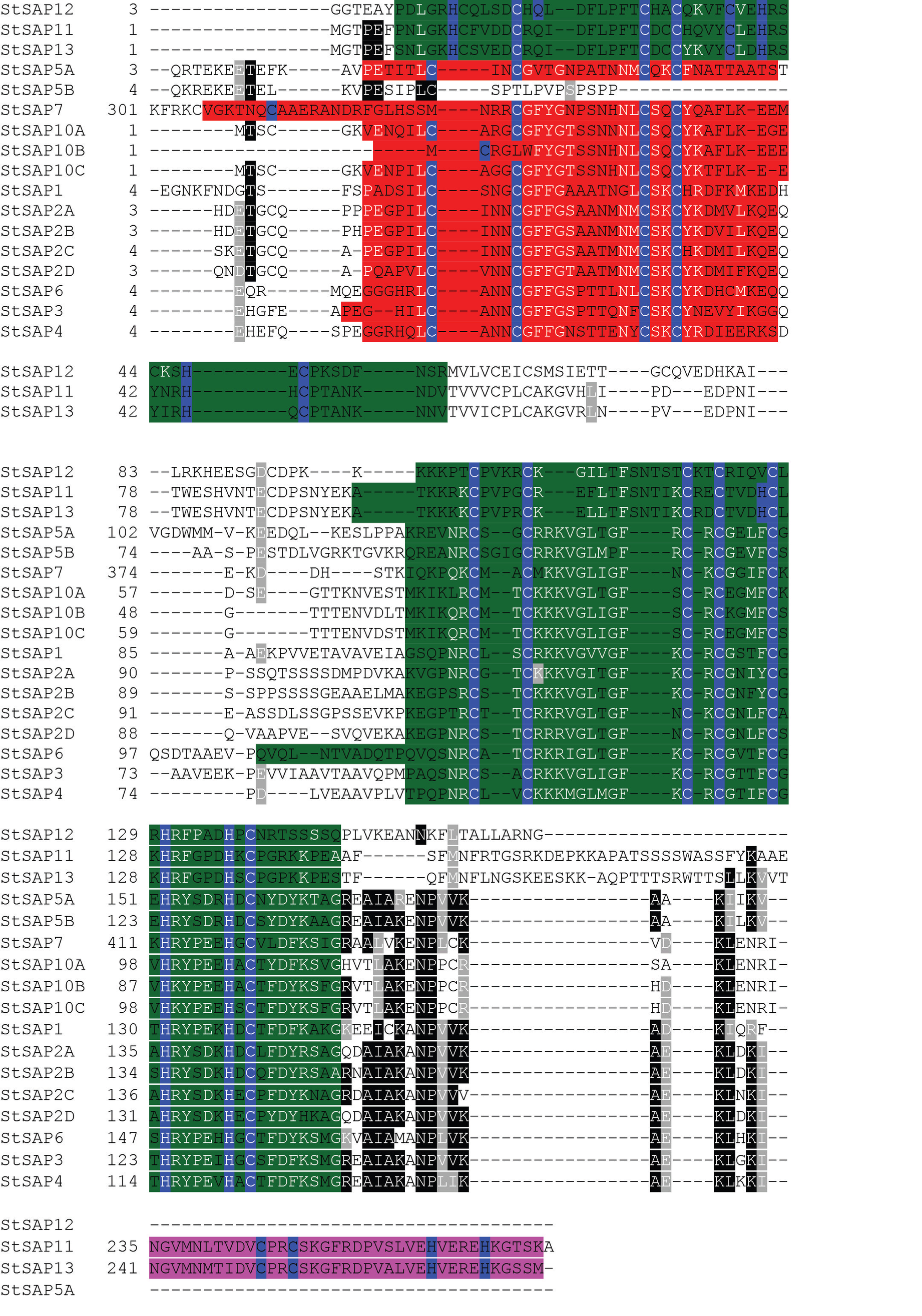

Supplement: S1 Fig — A20 zinc-finger domains are characterized by conserved cysteine residues and labelled Red, whereas the AN1 zinc-finger domain has conserved histidine and cysteine residues and colored Green. Proteins that had C2H2 zinc-finger domains were colored Pink. (TIF) [file pone.0273416.s001.tif]

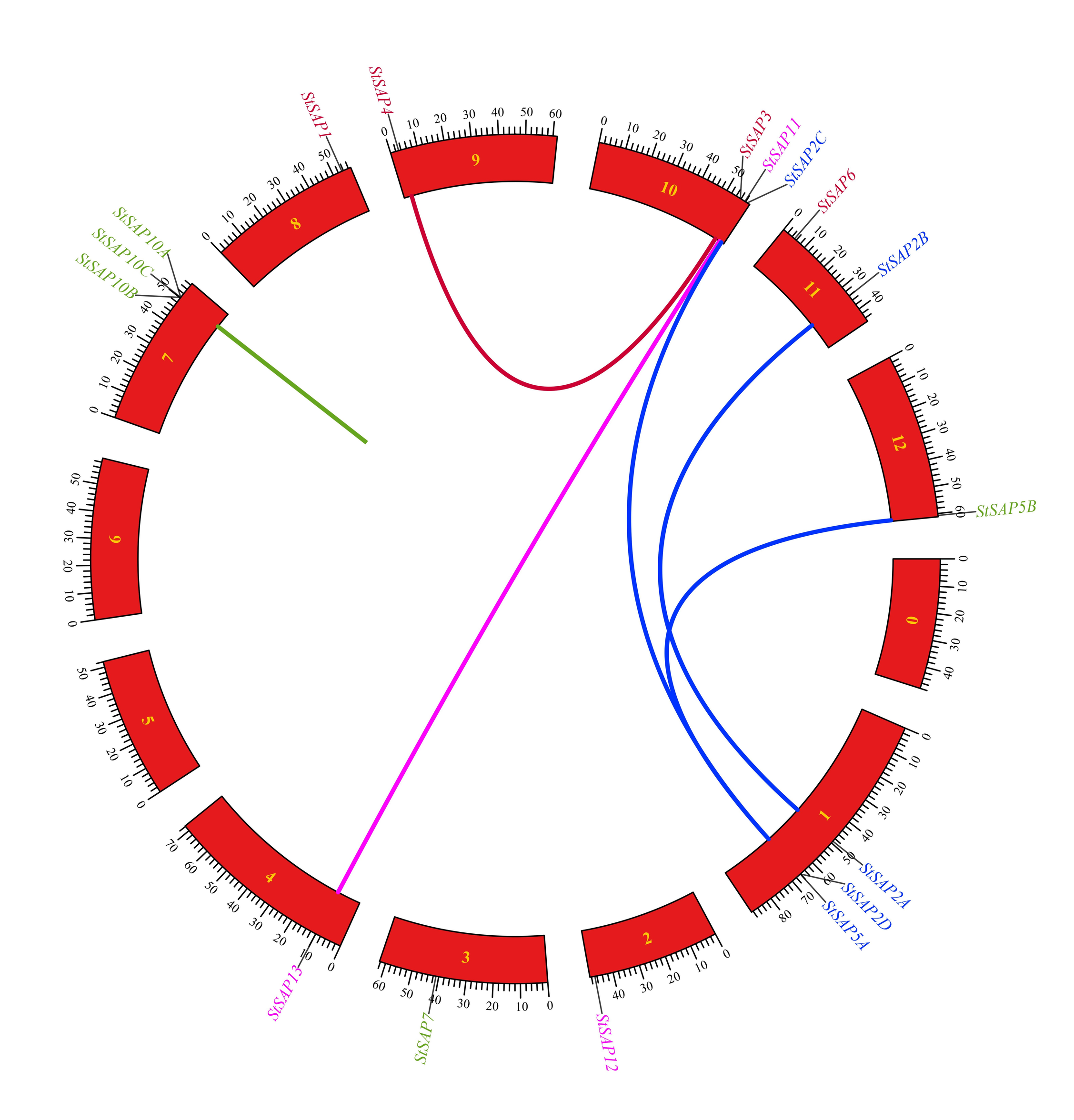

Supplement: S2 Fig — (TIF) [file pone.0273416.s002.tif]
